# CDK1 Phosphorylates KAT8 at Ser348 to Stabilize the MSL Complex and Promote H4K16 Acetylation in Non-Small Cell Lung Cancer

**DOI:** 10.3390/cells15100897

**Published:** 2026-05-14

**Authors:** Jinmeng Chu, Qingzhi Zhao, Hui Ye, Meixu Li, Yizhen Wang, Tiantian Xu, Yong Cai, Jingji Jin

**Affiliations:** School of Life Sciences, Jilin University, Changchun 130012, China; chujm21@mails.jlu.edu.cn (J.C.); zhaoqz23@mails.jlu.edu.cn (Q.Z.); yehuiswx@163.com (H.Y.); mx953665@126.com (M.L.); yizhen23@mails.jlu.edu.cn (Y.W.); xutt23@mails.jlu.edu.cn (T.X.); caiyong62@jlu.edu.cn (Y.C.)

**Keywords:** KAT8, CDK1, phosphorylation, non-small cell lung cancer, drug-resistance, cell proliferation

## Abstract

**Highlights:**

**What are the main findings?**

- CDK1 directly phosphorylates KAT8 at Ser348 and Thr418, with Ser348 serving as the dominant regulatory site.
- CDK1-mediated phosphorylation, particularly at Ser348, stabilizes the MSL complex by enhancing KAT8-MSL1 interaction and promotes H4K16 acetylation.

**What are the implications of the main findings?**

- The phosphorylation-deficient KAT8-S348A mutant impairs H4K16 acetylation and suppresses NSCLC cell proliferation both in vitro and in vivo.
- Pharmacological inhibition of CDK1 with RO-3306 reduces KAT8 phosphorylation and tumor growth, identifying the CDK1–KAT8 axis as a potential therapeutic target in lung cancer.

**Abstract:**

Cyclin-dependent kinase 1 (CDK1) is frequently upregulated in multiple cancers and plays a central role in cell cycle progression and tumorigenesis. However, whether CDK1 directly regulates the histone acetyltransferase KAT8 (also known as MOF) in non-small cell lung cancer (NSCLC) remains unclear. Here, we identify CDK1 as a kinase that directly interacts with and phosphorylates KAT8 at serine 348 (S348) and threonine 418 (T418). Mechanistically, CDK1-mediated phosphorylation, particularly at S348, enhances the interaction between KAT8 and MSL1, thereby stabilizing the MSL complex and promoting KAT8-dependent acetylation of histone H4 at lysine 16 (H4K16). Functionally, the phosphorylation-deficient mutant KAT8-S348A exhibits impaired MSL complex assembly, reduced H4K16 acetylation, and decreased NSCLC cell proliferation both in vitro and in vivo. Pharmacological inhibition of CDK1 using RO-3306 suppresses KAT8 phosphorylation and H4K16 acetylation, leading to significant tumor growth inhibition. Notably, this effect is partially rescued by re-expression of wild-type KAT8 but not by the S348A mutant, supporting a phosphorylation-dependent mechanism. Collectively, these findings define a CDK1–KAT8 signaling axis that promotes NSCLC proliferation through epigenetic regulation and suggest that targeting CDK1-dependent KAT8 phosphorylation may represent a potential therapeutic strategy for lung cancer.

## 1. Introduction

Lung cancer remains one of the most lethal malignancies worldwide and is broadly classified into non-small cell lung cancer (NSCLC) and small cell lung cancer (SCLC) [1]. Surgical resection is the preferred treatment for patients with early-stage NSCLC, whereas advanced disease requires a comprehensive therapeutic strategy guided by histopathological subtype, tumor stage, molecular alterations, and patient condition. In this context, chemotherapy, radiotherapy, and molecularly targeted therapies, and immune checkpoint inhibitors constitute the principal treatment modalities [2]. In addition, emerging approaches, including natural product-derived compounds, are being actively explored as potential anti-lung cancer agents [3]. Despite substantial advances in targeted therapy and immunotherapy, disease recurrence, acquired resistance, and limited long-term survival remain major clinical challenges in NSCLC management. Therefore, identifying mechanistic vulnerabilities that connect cell cycle signaling with epigenetic regulation may offer additional therapeutic opportunities.

Cyclin-dependent kinases (CDKs) are frequently upregulated in human cancers and are closely associated with patient survival across multiple tumor types [4,5]. Among them, CDK1 plays a central and nonredundant role in cell cycle regulation and is strongly implicated in tumor initiation and progression [6]. Accumulating evidence indicates that diverse regulatory molecules modulate tumor growth, migration, invasion, and therapeutic resistance through CDK1-dependent mechanisms. For example, OTUD4 promotes glioblastoma invasion by deubiquitinating CDK1 and activating the MAPK signaling pathway [7], whereas KLF7 enhances lung squamous cell carcinoma progression by transactivating PPP1R14C to sustain CDK1 activity [7,8]. In addition, miR-31 suppresses lung adenocarcinoma cell proliferation by inducing CDK1- and E2F2-mediated cell cycle arrest [9], and CEP55 promotes cisplatin resistance in prostate cancer by regulating CDK1 phosphorylation [10]. CDK1 also facilitates leukemia progression by modulating the interaction between EZH2 (Enhancer of Zeste Homolog 2) and DNMT3A (DNA Methyltransferase 3 Alpha). Consistently, pharmacological inhibition of CDK1 with RO-3306 exhibits antitumor activity in high-grade serous ovarian cancer [11].

KAT8, also known as MOF or MYST1, is a member of the MYST family of histone acetyltransferases and was initially identified in *Drosophila* as a component of the male-specific lethal (MSL) complex involved in X chromosome dosage compensation [12,13]. KAT8 regulates diverse cellular processes, including gene transcription, DNA damage repair, chromatin stability, and mitochondrial homeostasis, by acetylating histone substrates (H4K5, H4K8, H4K16) as well as non-histone proteins [14]. Increasing evidence highlights a pivotal role for KAT8 and the MSL complex in tumorigenesis through its acetyltransferase activity. For instance, KAT8-mediated lactylation of NSUN2 promotes bladder cancer proliferation by modulating m5C modification of HELZ2 [15]. KAT8 also acetylates SEPP1 at lysine 247/249 to regulate CD8^+^ T cell activity via LRP8, thereby promoting antitumor immunity in pancreatic cancer [16], and enhances cisplatin resistance in lung cancer through acetylation of PKM2 [17]. In addition, KAT8 activity is tightly controlled by post-translational modifications; for example, SIRT1 negatively regulates its activity and protein stability [18].

Although PBK has been reported to phosphorylate MSL1 and thereby affects MSL complex stability [19], phosphorylation of KAT8 remains poorly characterized. In particular, how KAT8 phosphorylation regulates its enzymatic activity and the integrity of the MSL complex is still unknown. Previous studies have established a link between CDK1 and epigenetic regulation; however, whether CDK1 directly phosphorylates KAT8 and how this modification contributes to cancer progression remains unclear. In this study, we identify CDK1 as a kinase that phosphorylates KAT8, thereby enhancing MSL complex stability and acetyltransferase activity. Furthermore, inhibition of CDK1 suppresses lung cancer cell proliferation through modulation of KAT8 phosphorylation, highlighting a CDK1-KAT8 epigenetic regulatory axis as a potential therapeutic target in lung cancer.

## 2. Materials and Methods

### 2.1. Antibodies and Reagents

The Anti-Flag (M2) agarose, anti-Myc agarose, and anti-Flag M2 (F3165) mouse monoclonal antibody were obtained from Sigma-Aldrich (St. Louis, MO, USA). Anti-Myc (9E10) mouse monoclonal antibody was from Santa Cruz Biotechnology (Dallas, TX, USA). Anti-KAT8 (A3390), anti-CDK1 (AP0220), anti-CCNB1 (A16038), anti-Histone H4 (A23000) and anti-MSL1 (24373-1-AP) mouse monoclonal antibodies were from ABclonal Technology (Wuhan, China). Anti-H4K16ac (PTM-122) polyclonal antibody was from Jingjie Biotechnology (Hangzhou, China). Anti-HA (RLM3003) mouse monoclonal antibody was from Ruiying Biological (Suzhou, China). Anti-GFP (GB11602-100) rabbit polyclonal antibodies was from Epizyme Biotech (Shanghai, China). Anti-H3S10Ph (mouse monoclonal, #26436) was sourced from Upstate (New York, NY, USA). Anti-GST (BM3900) rabbit monoclonal antibody was provided by Boster Group (BM0101, Wuhan, China). Anti-Pho-Ser/Thr (9631S) polyclonal antibody was from Cell Signaling Technology (Boston, MA, USA). Anti-GAPDH (NM_002046, full length) rabbit polyclonal antibodies were raised against bacterially expressed proteins (Jilin University, Changchun, China).

KAT8 inhibitor MG149 (S7476) was from Selleck Chemicals (Shanghai, China). CDK1 inhibitor RO-3306 (872573-93-8) was from Abmole (Beijing, China). Hydroxyurea (HU, H8267) and nocodazole (M1404) were from Sigma (St. Louis, MO, USA).

### 2.2. Cell Culture

HEK293T, A549 and H1299 cells were obtained from the Type Culture Collection of the Chinese Academy of Sciences (Shanghai, China) and cells were maintained at 37 °C and 5% CO_2_ in Dulbecco’s modified Eagle’s medium (DMEM, Gibco, Life Technologies, Waltham, MA, USA), and cultured with 10% fetal bovine serum (FBS, Kang Yuan Biology, Tianjin, China) and 1% penicillin-streptomycin mixture (P/S, Thermo Fisher Scientific, Waltham, MA, USA).

### 2.3. Plasmid Construction and Transfection

The Full-length cDNAs encoding the human MSL1 (NM_001012241), KAT8 (NM_032188), CDK1 (NM_001786.5), different truncations including KAT8 (1-157,158-458 and 218-458 aa), different point mutants including KAT8 (S348A and T418A) were subcloned into a pcDNA3.1 (–) vector with Flag or Myc tags. The plasmids were transiently transfected into cells using polyethyleneimine (PEI) (23966, PolySciences, Beijing, China), according to the manufacturer’s instructions. All constructed plasmids were verified by full-length Sanger sequencing to confirm the presence of the intended mutations and the absence of unintended secondary mutations. Protein expression and the expected molecular weight were further confirmed by Western blot analysis following transient transfection into HEK293T cells.

### 2.4. Immunoprecipitation (IP)

HEK293T, A549 and H1299 cells cultured in 10-cm tissue culture dishes were used for transient transfection with the aforementioned Flag- or Myc-tagged plasmids. At 48 h post-transfection, the cells were harvested using RIPA lysis buffer composed of 1% NP-40, 150 mM NaCl, 50 mM Tris-HCl, 10% glycerol, 1 mM dithiothreitol (DTT), and a complete protease inhibitor cocktail. The resulting whole-cell lysates were incubated overnight at 4 °C with anti-Flag (M2) or anti-Myc agarose beads, or with Protein A beads pre-bound with antibodies. Immunoprecipitated proteins were subsequently eluted using 4× SDS sample buffer, and the captured proteins were analyzed by Western blotting using anti-Flag or anti-Myc antibodies.

### 2.5. Expression of Recombinant Proteins in Escherichia coli

Full-length or point mutants of CDK1, H4 and KAT8 were subcloned into pGEX-6p-1 vector. GST-tagged different proteins that were expressed from pGEX-6p-1 in BL21 (DE3) Codon Plus *E. coli* cells.

### 2.6. siRNA/shRNA Knockdown

The pLVX-shRNA system was used to express shRNA in A549 and H1299 cells, and the target sequences specific for CDK1 were used: shCDK1-1, GTGGAATCTTTACAGGACTAT; shCDK1-2, GTGGATGAAGATCCAACCCAA.

### 2.7. GST-Pulldown

After equilibrating the GST beads three times (2100 rpm, 6 min, 4 °C), an appropriate volume of the in vitro purified recombinant protein was added, and the mixture was incubated overnight with rotation on a mixer in a 4 °C cold room. Following elution, 50 μL of 4× SDS loading buffer was added, and the sample was boiled at 95 °C for 15 min.

### 2.8. Cell Counting-Kit-8 (CCK8)

After trypsinization and serial dilution for counting, approximately 2000 cells per well were seeded into a 96-well plate with three replicate wells per group and cultured in a 37 °C incubator. After cell attachment, 10 μL of CCK8 reagent (G3580, Promega Corporation, Madison, WI, USA) was added to each well at designated time points. Following incubation in a constant-temperature incubator for 1 h, the optical density (OD) value was measured at 450 nm using a microplate reader (Infinite F200 Pro, TECAN, Shanghai, China).

### 2.9. In Vitro Kinase Assay

Flag-CDK1 was transfected into HEK-293T cells. After 48 h, the CDK1-Cyclin B complex was enriched from whole-cell lysates by immunoprecipitation using Flag beads. The Flag bead-enriched CDK1-Cyclin B complex, along with Flag-KAT8 protein enriched from stable cell lines and GST protein, were incubated in kinase buffer (50 mM Tris-HCl, pH 7.4; 10 mM MgCl_2_; 2 mM EGTA; 100 mM KCl; 0.1 mM EDTA) with or without 1 mM ATP for 1 h at 37 °C. The reaction was terminated by adding 4× SDS loading buffer, and the samples were boiled at 95 °C for 15 min, followed by immunoblotting.

### 2.10. In Vitro HAT Assay

In vitro HAT assays were performed using recombinant histone H4 and Flag-KAT8, Flag-KAT8-S348A and Flag-KAT8-T418A purified from HEK293T cells. Purified proteins were incubated in HAT assay buffer (50 mM Tris–HCl, pH 8.0, 5% glycerol, 0.1 mM EDTA, 1 mM phenylmethylsulfonyl fluoride, 10 mM sodium butyrate, and 1 mM dithiothreitol) at 30 °C for 1 h in the presence of acetyl-CoA. The reaction was terminated by heating at 95 °C for 5 min in 4× SDS buffer, and the products were analyzed by Western blotting.

### 2.11. Immunofluorescence Staining

HEK293T cells were seeded onto coverslips (8D1007, Nest Biotechnology, Wuxi, China) in 24-well plates and cultured to 70–80% confluence. Cells were washed once with PBS and fixed with 4% paraformaldehyde for 15 min at room temperature. After a brief PBS wash, cells were permeabilized with 0.3% Triton X-100 for 10 min at room temperature. Nonspecific binding was blocked by incubating the cells with 1% BSA at 37 °C for 1 h. Cells were then incubated with the appropriate primary antibody at 37 °C for 2 h, followed by five washes with PBST. Secondary antibody incubation was performed at 37 °C for 1.5 h. Nuclear staining was conducted using DAPI (H-1200, Vector Laboratories, Inc., Burlingame, CA, USA). Fluorescent images were captured using fluorescence microscopes (Olympus BX40F, Olympus Corporation, Tokyo, Japan; and Carl Zeiss LSM700, Carl Zeiss AG, Oberkochen, Germany).

### 2.12. EdU Incorporation Assay

Cells were seeded onto coverslips for 24 h, then incubated with 10 μM EdU (5-ethynyl-2′-deoxyuridine) for 2 h in a 37 °C incubator. After fixation and permeabilization, the Click reaction was performed using the BeyoClick EdU-488 Kit (C0071S; Beyotime Biotechnology, Shanghai, China) for 30 min. Following washing with PBS, cell nuclei were stained with Hoechst dye. The coverslips were then mounted, and images were captured from three randomly selected regions per group under a microscope. The cell proliferation rate was subsequently calculated.

### 2.13. Colony Formation Assay

Stably transfected A549 and H1299 cell lines (3000–4000 cells/well) were seeded into 24-well plates and cultured for 10–14 days at 37 °C. Colonies were then fixed with 4% paraformaldehyde for 15 min and stained with 0.1% crystal violet for 20 min. The number and size of colonies were recorded for comparison and imaged using a digital camera.

### 2.14. Animal Experiments

Animal studies were approved by the Institutional Animal Care and Use Committee (IACUC) of Jilin University (Approval No. IACUC Issue No. SY: 2026-03-013; Approval date: 6 March 2026). Female Balb/c nude mice (4–5 weeks old, weighing 18–20 g) were purchased from SPF (Beijing) Biotechnology Co., Ltd. (Beijing, China). Mice were housed under a 12 h light/dark cycle with ad libitum access to food and water. Sample sizes were determined using the Resource Equation method. Randomization was performed based on body weight to ensure comparable baseline characteristic among groups. Stably transfected H1299 cells expressing Flag-vector, Flag-KAT8-WT, and Flag-KAT8-S348A were cultured to logarithmic growth phase and then subcutaneously injected into the right axilla of nude mice. Each mouse received 1 × 10^7^ cells. Mice were divided into six groups (*n* = 5 per group): vector control, KAT8-WT, KAT8-S348A, vector + RO-3306, KAT8-WT + RO-3306, and KAT8-S348A + RO-3306. When the tumor volume reached approximately 80–100 mm^3^, mice were administered intraperitoneal injections of either saline or RO-3306 (2 mg/kg) on days 1, 4, 7, 11, 14, 17, and 21. Tumor growth was monitored throughout the experimental period.

### 2.15. Statistical Analysis

Statistical analyses were conducted using data from at least three independent experiments. Data were processed with SPSS software, version 26 (IBM Corp., Armonk, NY, USA). Results are presented as mean ± SD. Differences between two groups were evaluated using an unpaired Student’s *t*-test, while comparisons among multiple groups were analyzed using one-way analysis of variance (ANOVA). A *p*-value < 0.05 was considered statistically significant.

## 3. Results

### 3.1. CDK1 Directly Phosphorylates KAT8 in a Cell Cycle-Dependent Manner

**Figure 1:**
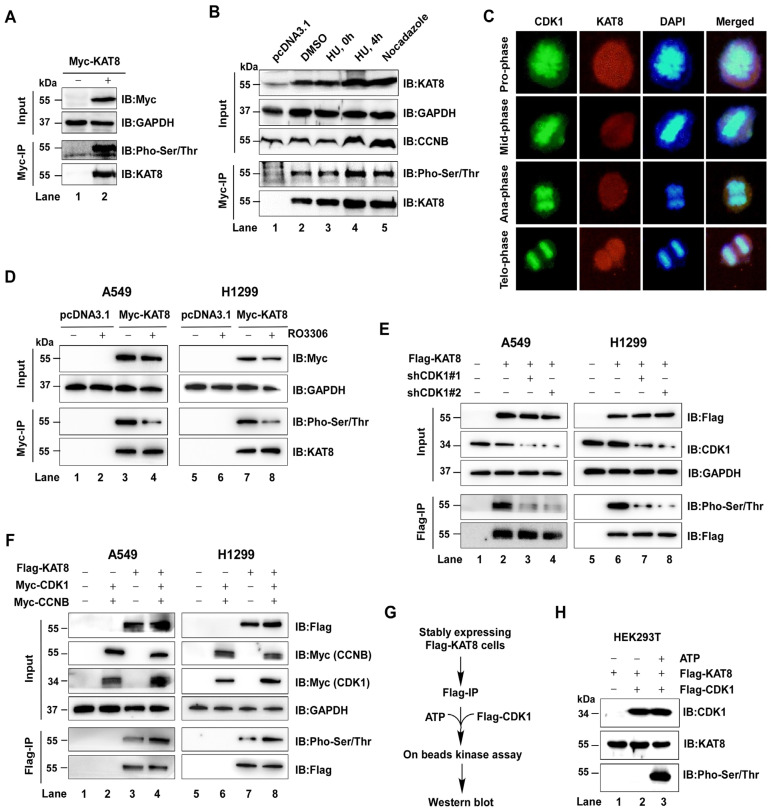
CDK1 phosphorylates KAT8. (**A**) Myc-tagged KAT8 was overexpressed in HEK293T cells, and interacting proteins were analyzed by immunoblotting following Myc immunoprecipitation. (**B**) HEK293T cells were treated with DMSO or hydroxyurea (HU) for 12 h, followed by release for 4 h, or treated with nocodazole for 12 h to enrich cells at distinct cell cycle stages (G1, S, and G2/M). KAT8 was immunoprecipitated, and phosphorylation was assessed using a pan-phospho antibody. (**C**) Endogenous CDK1 and KAT8 were detected by immunofluorescence. CDK1, green; KAT8, red. (**D**) Myc-KAT8 was overexpressed in A549 or H1299 cells and treated with the CDK1 inhibitor RO-3306 (2 μM, 12 h), followed by analysis of KAT8 phosphorylation. (**E**) Flag-KAT8 was overexpressed in A549 or H1299 cells with CDK1 knockdown, and phosphorylation was assessed by Flag immunoprecipitation and immunoblotting. (**F**) Flag-KAT8 was co-expressed with CDK1 and CCNB1 in A549 or H1299 cells, and phosphorylation was analyzed by immunoprecipitation and immunoblotting. (**G**) CDK1-Cyclin B complexes were purified from HEK293T cells by Flag immunoprecipitation and incubated with purified Flag-KAT8 in an in vitro kinase assay. (**H**) Immunoblot analysis showing that CDK1 directly phosphorylates KAT8 in vitro.

ATM has been reported to phosphorylate KAT8 at T392, thereby influencing DNA damage repair. However, whether KAT8 is regulated by additional kinases remains unclear [20]. To address this, we first confirmed that KAT8 is phosphorylated in HEK293T cells, consistent with previous findings (Figure 1A). Notably, KAT8 phosphorylation exhibited cell cycle-dependent dynamics, with increased levels during the S and G2/M phases (Figure 1B), suggesting regulation by cell cycle-associated kinases.

Given its central role in cell cycle progression, CDK1 was considered a potential upstream regulator. Immunofluorescence analysis revealed co-localization of CDK1 and KAT8 throughout the cell cycle (Figure 1C). To further examine this relationship, Myc-KAT8 was overexpressed in A549 and H1299 cells, followed by immunoprecipitation and detection with a pan-phospho antibody. Pharmacological inhibition of CDK1 using RO-3306 markedly reduced KAT8 phosphorylation (Figure 1D), an effect that was recapitulated by CDK1 knockdown (Figure 1E). Conversely, co-expression of CDK1 and its regulatory partner CCNB1 enhanced KAT8 phosphorylation (Figure 1F). To determine whether CDK1 directly phosphorylates KAT8, in vitro kinase assays were performed using purified CDK1-CCNB1 complex and purified Flag-KAT8 (Figure 1G). These assays demonstrated that CDK1 directly phosphorylates KAT8 in vitro (Figure 1H). Collectively, these findings identify CDK1 as a previously unrecognized kinase that directly phosphorylates KAT8 in a cell cycle-dependent manner.

### 3.2. CDK1 Directly Interacts with KAT8 via Its C-Terminal Domain

**Figure 2:**
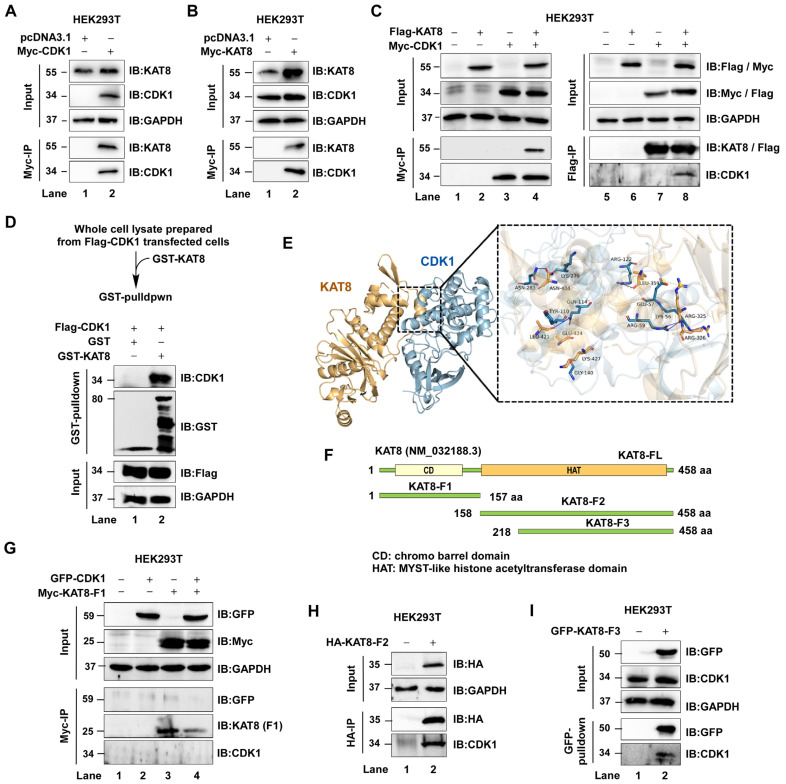
CDK1 directly interacts with KAT8. (**A**,**B**) Myc-tagged CDK1 or KAT8 was overexpressed in HEK293T cells, followed by Myc immunoprecipitation and immunoblot analysis of interacting proteins. (**C**) Flag-KAT8 and Myc-CDK1 were co-expressed, and their interaction was assessed by reciprocal immunoprecipitation followed by immunoblotting. (**D**) GST-tagged KAT8 was expressed in *E. coli* and purified for GST pull-down assays. CDK1 was overexpressed in HEK293T cells and enriched by immunoprecipitation. (**E**) Structural modeling of the CDK1–KAT8 interaction was performed using HDOCK. (**F**) Schematic of KAT8 truncation mutants: KAT8-F1 (amino acids 1–157), KAT8-L2 (158–458), and KAT8-L3 (218–458). (**G**) EGFP-CDK1 was co-expressed with KAT8-F1, and interaction was assessed by immunoprecipitation. (**H**) HA-KAT8-L2 was overexpressed in HEK293T cells, followed by HA immunoprecipitation and detection of endogenous CDK1. (**I**) Interaction between CDK1 and the KAT8-L3 truncation mutant was assessed by immunoprecipitation.

Given that CDK1 phosphorylates KAT8, we next examined whether these proteins physically associate. Myc-CDK1 or Myc-KAT8 was overexpressed in HEK293T cells, followed by immunoprecipitation and immunoblotting. Myc-CDK1 was found to associate with endogenous KAT8, and conversely, Myc-KAT8 co-precipitated with endogenous CDK1 (Figure 2A,B). Co-expression of Flag-CDK1 and Myc-KAT8 further confirmed this interaction at the exogenous level (Figure 2C).

To determine whether this interaction is direct, GST pull-down assays were performed using purified GST-KAT8, which demonstrated a direct interaction between KAT8 and CDK1 (Figure 2D). Structural modeling using HDOCK predicted that the C-terminal region of KAT8 mediates CDK1 binding (Figure 2E). To experimentally validate this prediction, a series of KAT8 truncation mutants was generated: KAT8-F1 (amino acids 1–157), KAT8-L2 (158–458), and KAT8-L3 (218–458) (Figure 2F). Pull-down assays demonstrated that KAT8-F1 failed to interact with CDK1, whereas both KAT8-L2 and KAT8-L3 retained binding capacity (Figure 2G–I), indicating that the C-terminal region (aa 218–458) is sufficient for CDK1 interaction. Together, these results establish that CDK1 directly interacts with KAT8 through its C-terminal domain.

### 3.3. CDK1 Phosphorylates KAT8 at S348 and T418

**Figure 3:**
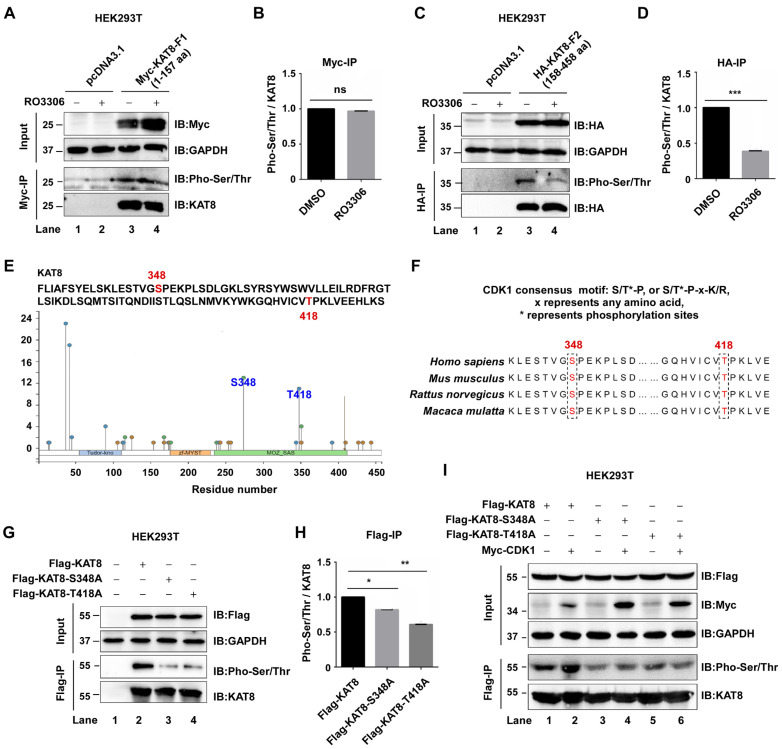
CDK1 phosphorylates KAT8 at S348 and T418. (**A**) Myc-KAT8-F1 was expressed in HEK293T cells in the presence or absence of the CDK1 inhibitor RO-3306 (2 μM, 12 h), and phosphorylation was detected using a pan-phospho antibody. (**B**) Quantification of phosphorylation levels relative to total KAT8 in (**A**). (**C**) Phosphorylation of the KAT8-L2 truncation mutant following treatment with RO-3306 treatment (2 μM, 12 h). (**D**) Quantification of phosphorylation levels relative to total KAT8 in (**C**). (**E**) Identification of putative CDK1 phosphorylation sites (S348 and T418) within KAT8 based on consensus motif analysis and PhosphoSitePlus predictions. (**F**) Sequence alignment showing conservation of S348 and T418 across species. (**G**) Wild-type KAT8, KAT8-S348A, or KAT8-T418A mutants were expressed in HEK293T cells, and phosphorylation was assessed by immunoprecipitation followed by immunoblotting. (**H**) Quantification of phosphorylation levels relative to total KAT8 in (**G**). (**I**) Wild-type KAT8 or phosphorylation-deficient mutants (S348A and T418A) were co-expressed with or without CDK1, and phosphorylation levels were analyzed by Western blot using specific antibodies. Statistical annotations: ns, not significant; * *p* < 0.05; ** *p* < 0.01; *** *p* < 0.001.

Having established that CDK1 interacts with and phosphorylates KAT8, we next sought to map the specific phosphorylation sites. To localize the CDK1-responsive region, KAT8 truncation mutants were generated and analyzed. HEK293T cells expressing Myc-tagged KAT8-F1 (aa 1–157) or HA-tagged KAT8-L2 (aa 158–458) were treated with the CDK1 inhibitor RO-3306, followed by immunoprecipitation and immunoblot analysis. CDK1 inhibition had no detectable effect on phosphorylation of the F1 fragment, whereas phosphorylation of the L2 fragment was markedly reduced (Figure 3A–D), indicating that CDK1-responsive phosphorylation sites are located within the L2 region.

CDK1 is a proline-directed kinase that preferentially recognizes the minimal consensus motif S/T-P. Based on this motif and in silico predictions using PhosphoSitePlus (www.phosphosite.org; accessed on 13 May 2025), two candidate residues within the L2 region, S348 and T418, were identified (Figure 3E,F). Sequence alignment across multiple species (human, mouse, rat, and rhesus monkey) further revealed that both residues are highly conserved (Figure 3F). To validate these sites experimentally, alanine substitution mutants of KAT8 (S348A and T418A) were generated. Compared with wild-type KAT8, both mutants exhibited significantly reduced phosphorylation levels (Figure 3G,H). Consistently, CDK1 overexpression enhanced phosphorylation of wild-type KAT8 but failed to further increase phosphorylation of either mutant (Figure 3I). Collectively, these data identify S348 and T418 as two major CDK1 phosphorylation sites on KAT8.

### 3.4. CDK1-Mediated Phosphorylation of KAT8 Enhances MSL Complex Stability and H4K16 Acetylation

**Figure 4:**
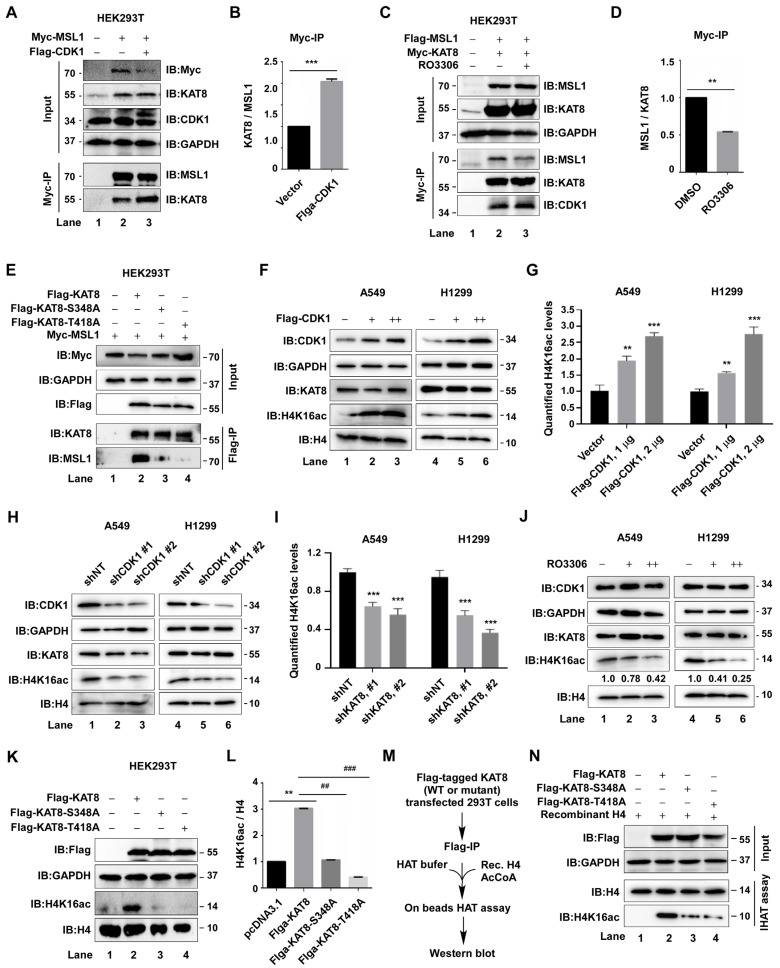
CDK1-mediated KAT8 phosphorylation of KAT8 promotes MSL complex assembly and acetyltransferase activity. (**A**) Myc-MSL1 and Flag-CDK1 were co-expressed in HEK293T cells, followed by immunoprecipitation of MSL1 and detection of associated endogenous KAT8. (**B**) Quantification of KAT8 levels relative to MSL1 in (**A**). (**C**) Flag-MSL1 and Myc-KAT8 were co-expressed with or without RO-3306 (2 μM, 12 h), and interactions were assessed by immunoprecipitation and immunoblotting. (**D**) Quantification of MSL1 levels relative to KAT8 in (**C**). (**E**) Wild-type KAT8 or phosphorylation-deficient mutants (S348A and T418A) were co-expressed with Myc-MSL1, and binding to MSL1 was assessed. (**F**) CDK1 was expressed at increasing levels in A549 or H1299 cells, and H4K16 acetylation was analyzed by immunoblotting. (**G**) Quantification of H4K16ac levels normalized to loading controls in (**F**). (**H**) H4K16 acetylation levels following CDK1 knockdown in A549 or H1299 cells. (**I**) Quantification of H4K16ac levels in (**H**). (**J**) A549 or H1299 cells were treated with RO-3306 (1 or 2 μM, 12 h), and H4K16 acetylation was assessed. (**K**) Wild-type KAT8 or phosphorylation-deficient mutants were expressed in HEK293T cells, and global H4K16 acetylation levels were analyzed. (**L**) Quantification of H4K16ac levels normalized to total H4 protein in (**K**). (**M**) Flag-tagged wild-type KAT8 or mutants were immunoprecipitated and subjected to in vitro acetylation assays using purified histone H4 substrate. (**N**) Quantification of H4K16 acetylation in (**M**), showing reduced acetyltransferase activity of phosphorylation-deficient mutants. Statistical annotations: ** *p* < 0.01 and *** *p* < 0.001 vs. the indicated control; ## *p* < 0.01 and ### *p* < 0.001 vs. the indicated comparison group.

KAT8 functions as a histone acetyltransferase primarily within the MSL and NSL multiprotein complexes. We first examined whether CDK1 regulates MSL complex assembly. CDK1 overexpression enhanced the interaction between KAT8 and the core MSL component MSL1 (Figure 4A,B), whereas pharmacological inhibition of CDK1 with RO-3306 reduced this interaction (Figure 4C,D). Consistently, alanine substitution of the CDK1 phosphorylation sites S348 and T418 diminished KAT8-MSL1 binding (Figure 4E), indicating that CDK1-mediated phosphorylation contributes to MSL complex stability. Given that the MSL complex is responsible for catalyzing histone H4 lysine 16 acetylation (H4K16ac), we next assessed whether CDK1 regulates this epigenetic mark. CDK1 overexpression increased global H4K16 acetylation in A549 and H1299 cells, whereas CDK1 knockdown or RO-3306 treatment produced the opposite effect (Figure 4F–J), suggesting that CDK1 modulates H4K16ac through KAT8 regulation.

To directly evaluate the functional role of phosphorylation, wild-type KAT8 or phosphorylation-deficient mutants (S348A and T418A) were overexpressed. Compared with wild-type KAT8, both mutants exhibited markedly reduced ability to increase H4K16 acetylation (Figure 4K,L). Consistently, in vitro acetyltransferase assays demonstrated significantly decreased enzymatic activity of the mutants relative to wild-type KAT8 (Figure 4M,N). Together, these data demonstrate that CDK1-mediated phosphorylation of KAT8 at S348 and T418 promotes MSL complex stability and enhances KAT8 acetyltransferase activity, thereby increasing H4K16 acetylation.

### 3.5. KAT8 S348 Phosphorylation Is Required for Lung Cancer Cell Proliferation

**Figure 5:**
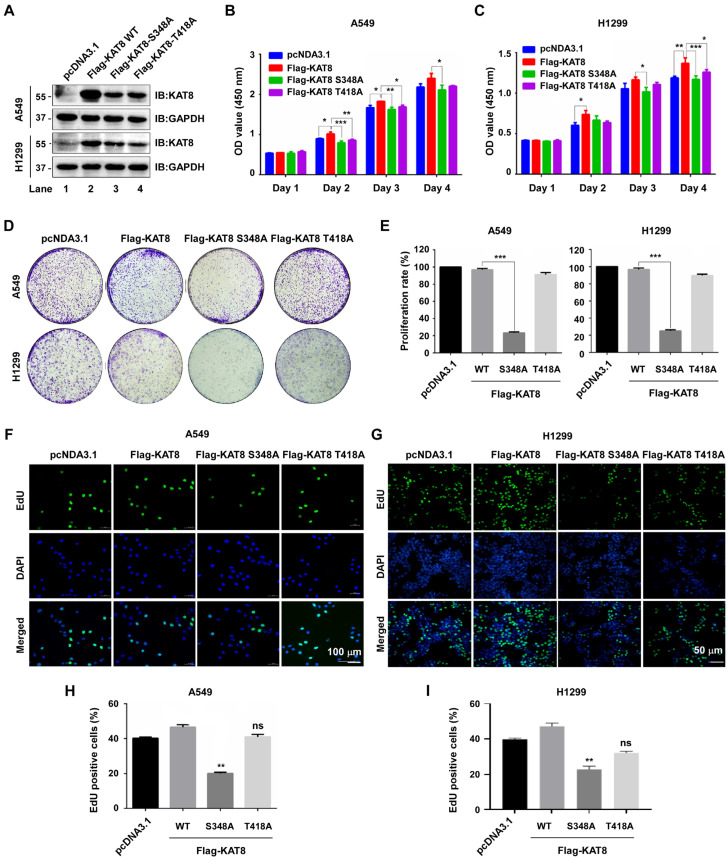
CDK1-mediated KAT8 phosphorylation of KAT8 promotes lung cancer cell proliferation. (**A**) Stable A549 and H1299 cell lines expressing wild-type KAT8 or phosphorylation-deficient mutants (S348A and T418A) were established. (**B**,**C**) Cell viability was assessed using CCK8 assays. (**D**) Colony formation assays were performed to evaluate proliferative capacity. (**E**) Quantification of colony formation in (**D**). (**F**) Proliferation of A549 stable cells was assessed by EdU incorporation. EdU-positive cells (green) and DAPI-stained nuclei (blue) are shown. (**G**) EdU incorporation assays in H1299 cells showing enhanced proliferation with wild-type KAT8, reduced proliferation with the S348 mutant, and no significant change with the T418A. (**H**) Quantification of EdU-positive A549 cells in (**F**). (**I**) Quantification of EdU-positive H1299 cells in (**G**). Statistical annotations: * *p* < 0.05; ** *p* < 0.01; *** *p* < 0.001.

CDK1-mediated phosphorylation enhances KAT8 acetyltransferase activity. Given the frequent dysregulation of KAT8 in cancer, we next investigated the functional relevance of its phosphorylation in lung cancer cells. Stable A549 and H1299 cell lines expressing Flag-tagged wild-type KAT8, or the phosphorylation-deficient mutants (S348A and T418A) were established (Figure 5A). CCK8 assays showed that both S348A and T418A mutants reduced cell viability compared with wild-type KAT8 (Figure 5B,C). However, more proliferation specific assays revealed a clear functional divergence between the two sites. Colony formation assays demonstrated that the S348A mutant markedly impaired clonogenic capacity, whereas the T418A mutant showed only a modest effect (Figure 5D,E). Consistently, EdU incorporation assays confirmed that mutation of S348, but not T418, significantly suppressed DNA synthesis and proliferative activity relative to wild-type KAT8 (Figure 5F–I). Collectively, these results indicate that phosphorylation of KAT8 at S348 is the dominant regulatory event required to sustain lung cancer cell proliferation, whereas T418 plays a more limited or auxiliary role.

### 3.6. CDK1 Is Upregulated in NSCLC and Its Pharmacological Inhibition Suppresses Tumor Cell Proliferation

**Figure 6:**
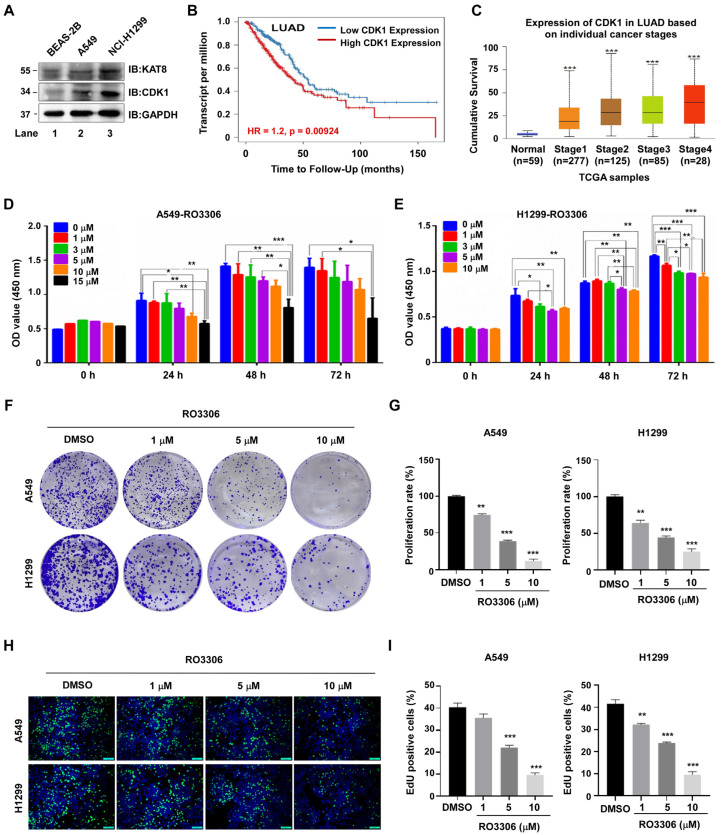
Pharmacological inhibition of CDK1 suppresses lung cancer cell proliferation. (**A**) Whole-cell lysates from NSCLC cell lines (A549 and H1299) and normal lung epithelial cells (BEAS-2B) were analyzed by immunoblotting for CDK1 and KAT8 expression. (**B**) Association between CDK1 expression and overall survival in lung adenocarcinoma patients analyzed using the TIMER2.0 database. (**C**) Analysis of CDK1 expression across clinical stages of lung adenocarcinoma using the UALCAN database. (**D**,**E**) A549 or H1299 cells were treated with increasing concentrations of the CDK1 inhibitor RO-3306, and cell viability was assessed using CCK8 assays. (**F**) Colony formation assays assessing the effect of RO-3306 on proliferative capacity. (**G**) Quantification of colony formation in (**F**). (**H**) EdU incorporation assays evaluating proliferation following RO-3306 treatment in A549 and H1299 cells. EdU-positive cells (green) and DAPI-stained nuclei (blue) are shown. (**I**) Quantification of EdU-positive cells in (H). Statistical annotations: * *p* < 0.05; ** *p* < 0.01; *** *p* < 0.001.

Aberrant CDK1 expression disrupts cell cycle homeostasis and contributes to tumor progression. Compared with normal lung epithelial BEAS-2B cells, both A549 and H1299 lung cancer cells exhibited elevated expression of KAT8 and CDK1 (Figure 6A), consistent with a potential coordinated dysregulation of this signaling axis in NSCLC. Analysis of the TIMER2.0 database further showed that low CDK1 expression was significantly associated with improved overall survival in lung adenocarcinoma patients (*p* = 0.00924) (Figure 6B). In addition, UALCAN database analysis revealed a progressive increase in CDK1 expression across clinical stages I–IV (*p* < 0.001) (Figure 6C).

Given the elevated expression of CDK1 in NSCLC, we next evaluated the functional impact of this inhibition. Treatment of A549 and H1299 cells with the selective CDK1 inhibitor RO-3306 resulted in a dose-dependent decrease in cell viability (Figure 6D,E), which was further confirmed by colony formation assays (Figure 6F,G) and EdU incorporation assays (Figure 6H,I). Together, these results suggest that CDK1 is functionally required for lung cancer cell proliferation. In the context of our mechanistic findings, this proliferative dependency is consistent with CDK1 acting, at least in part, through regulation of KAT8 phosphorylation and downstream epigenetic control. These data further support CDK1 as a potential therapeutic target in NSCLC.

### 3.7. RO-3306 Suppresses Lung Cancer Cell Proliferation by Targeting CDK1-Dependent KAT8 Phosphorylation

**Figure 7:**
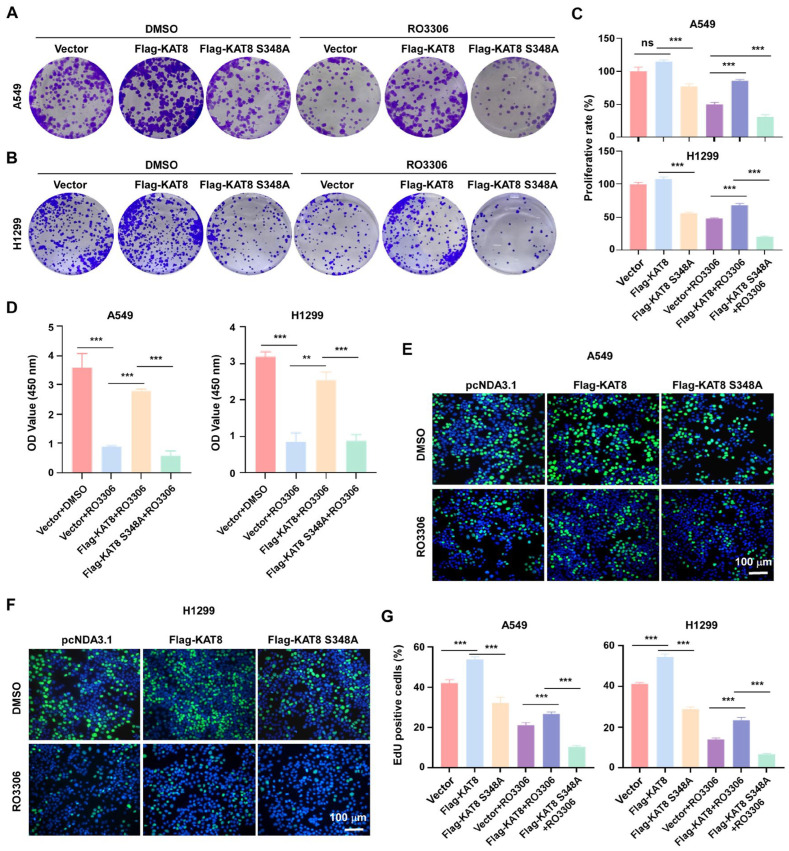
RO-3306 suppresses lung cancer cell proliferation through inhibition of KAT8 phosphorylation. (**A**) Colony formation assays in A549 (**A**) and H1299 (**B**) cells expressing wild-type KAT8 or the S348A mutant, with or without RO-3306 treatment. (**C**) Quantification of colony formation in (**A**). (**D**) Cell viability assessed by CCK8 assays following RO-3306 treatment. (**E**) EdU incorporation assays in A549 (**E**) and H1299 (**F**) cells expressing wild-type KAT8 or the S348A mutant, with or without RO-3306 treatment. EdU-positive cells (green) and DAPI-stained nuclei (blue) are shown. (**G**) Quantification of EdU-positive cells in (**E**) (left) and (**F**) (right). Statistical annotations: ns, not significant; ** *p* < 0.01; *** *p* < 0.001.

RO-3306 inhibits proliferation of A549 and H1299 lung cancer cells. To determine whether this effect involves CDK1-mediated phosphorylation of KAT8, cells expressing either wild-type KAT8 or the phosphorylation-deficient mutant KAT8-S348A were treated with RO-3306 and assessed using colony formation, CCK8, and EdU incorporation assays. In colony formation assays, overexpression of wild-type KAT8 partially attenuated the anti-proliferative effect of RO-3306, whereas cells expressing KAT8-S348A failed to rescue clonogenic growth and remained highly sensitive to CDK1 inhibition (Figure 7A–C). Consistently, CCK8 and EdU incorporation assays demonstrated that wild-type KAT8 partially restored cell viability and DNA synthesis under RO-3306 treatment, whereas the S348A mutant did not (Figure 7D–G). Collectively, these allele-specific rescue experiments indicate that CDK1 inhibition suppresses lung cancer cell proliferation in part by disrupting KAT8 phosphorylation at S348, thereby impairing its pro-proliferative acetyltransferase function. These findings position KAT8 as a key functional effector downstream of CDK1 signaling in NSCLC.

### 3.8. Pharmacological Inhibition of CDK1 by RO-3306 Suppresses Lung Tumor Growth In Vivo in a KAT8 S348-Dependent Manner

**Figure 8:**
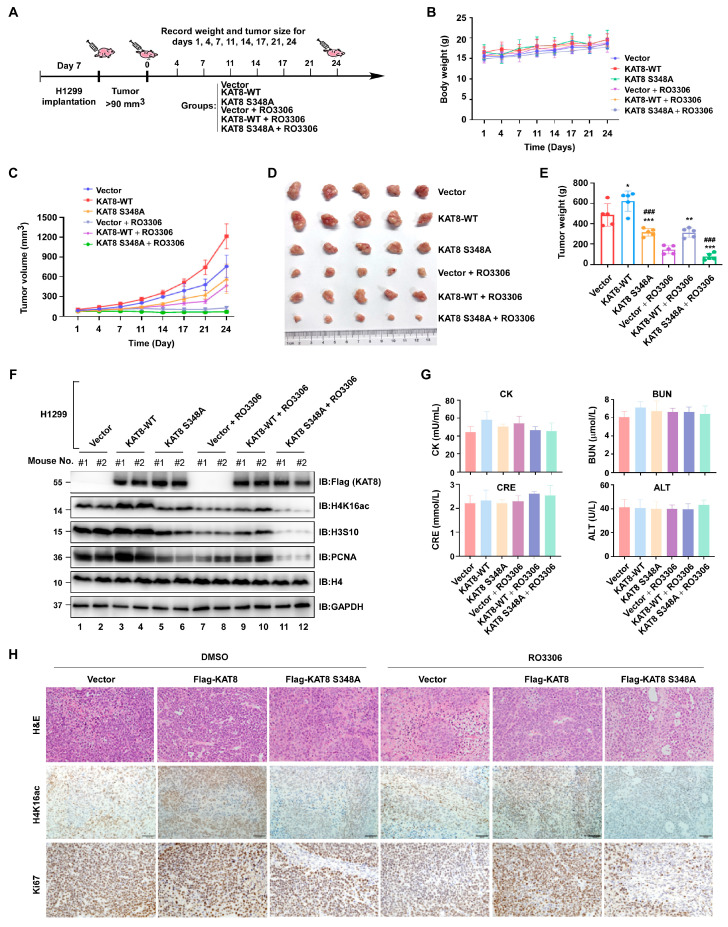
RO-3306 suppresses lung tumor growth in vivo by inhibiting CDK1-mediated KAT8 phosphorylation. (**A**) Schematic of subcutaneous xenograft model and treatment regimen. (**B**) Body weight monitored throughout the treatment period. (**C**) Tumor growth curves shown as mean ± SD. (**D**) Representative images of excised tumors at endpoint. (**E**) Tumor weights at endpoint. (**F**) Western blot analysis of protein expression in tumors lysates. (**G**) Serum biochemical analysis (CK, BUN, CRE, ALT). (**H**) Representative H&E staining and immunohistochemical staining for H4K16ac and Ki67 in tumor tissues. Statistical annotations: * *p* < 0.05; ** *p* < 0.01; *** *p* < 0.001 versus the indicated control; ### *p* < 0.001 vs. the indicated comparison group.

To evaluate the functional relevance of the CDK1–KAT8 axis in vivo, xenograft assays were performed using 5-week-old nude mice. Mice were randomly assigned to six groups: Vector + saline, KAT8 WT + saline, KAT8-S348A + saline, Vector + RO-3306, KAT8 WT + RO-3306, and KAT8-S348A + RO-3306. H1299 cells (1 × 10^7^) were subcutaneously injected into the axillary region, and when tumors reached approximately 80–100 mm^3^, mice were administered intraperitoneal injections of saline or RO-3306 (2 mg/kg, intraperitoneally) on days 1, 4, 7, 11, 14, 17, and 21 (Figure 8A). Body weights remained stable throughout the treatment, indicating good tolerability (Figure 8B). RO-3306 treatment significantly suppressed tumor growth in vivo. Overexpression of wild-type KAT8 partially attenuated this inhibitory effect, whereas expression of the phosphorylation-deficient KAT8-S348A mutant further enhanced tumor suppression. Notably, KAT8-S348A-expressing tumors were less responsive to KAT8-mediated rescue under CDK1 inhibition (Figure 8C–E), suggesting a phosphorylation-dependent requirement of KAT8 for tumor progression.

Consistently, immunoblot assays of tumor lysates revealed that both S348A expression and RO-3306 treatment reduced H4K16 acetylation, along with decreased levels of proliferation-associated markers PCNA and phospho-H3S10. These effects were partially reversed by wild-type KAT8 overexpression (Figure 8F), further supporting the functional link between CDK1 activity, KAT8 phosphorylation, and epigenetic regulation in vivo. Serum biochemical parameters, including creatine kinase (CK), creatinine (CRE), blood urea nitrogen (BUN), and alanine aminotransferase (ALT), showed no significant differences among treatment groups (Figure 8G), and histopathological evaluation by H&E staining revealed marked histological disruption in tumors from RO-3306-treated and KAT8-S348A + RO-3306 groups (Figure 8H, upper panel). Immunohistochemistry confirmed reduced Ki67 staining and decreased H4K16ac levels in these tumors (Figure 8H, middle and lower panel). Collectively, these in vivo data support a model in which CDK1 inhibition suppresses lung tumor growth, at least in part, through disruption of KAT8 S348 phosphorylation and downstream attenuation of H4K16ac, linking CDK1-driven epigenetic regulation to tumor proliferation.

We acknowledge that the current toxicological assessment is limited to routine serum biochemical parameters and histological evaluation. Comprehensive safety profiling, including hematological analysis and multi-organ histopathology, will be required to fully evaluate the translational potential of this strategy.

## 4. Discussion

In this study, we demonstrate that CDK1 phosphorylates KAT8 at residues S348 and T418, supported by complementary evidence including site-directed mutagenesis, loss of phosphorylation signals in corresponding mutants, and associated functional phenotypes. These approaches are widely accepted for inferring phosphorylation events in the absence of site-specific phospho-antibodies or mass spectrometry validation. Mechanistically, phosphorylation at S348 enhances KAT8–MSL1 interaction, stabilizes the MSL complex, and potentiates KAT8 acetyltransferase activity, leading to increased H4K16 acetylation. In NSCLC, where CDK1 is frequently overexpressed, pharmacological inhibition with RO-3306 reduces KAT8 phosphorylation and suppresses tumor cell proliferation both in vitro and in vivo.

KAT8 regulates diverse cellular processes, including proliferation, DNA repair, transcription, autophagy, and tumor invasion [20,21,22], and its post-translational modifications critically modulate its activity. For example, autoacetylation at K274 is required for enzymatic activity [23], whereas SIRT1-mediated deacetylation at this site influences chromatin recruitment and stability [24]. Additional modifications, including GCN5-mediated acetylation [25], MSL2-dependent ubiquitinates [26], and ATM-mediated phosphorylates at T392 [20], further regulate KAT8 stability, localization, and DNA repair function. Our findings add CDK1-mediated phosphorylation to this regulatory network. Among the two phosphorylation sites identified, S348 appears to be the dominant functional residue. Mutation of S348 markedly disrupts KAT8-MSL1 interaction, reduces MSL complex stability, diminishes H4K16ac levels, and impairs proliferative phenotypes. In contrast, T418 mutation exerts a comparatively modest effect on H4K16ac and does not fully phenocopy the proliferative defect observed with S348A. These observations support a hierarchical model in which S348 phosphorylation primarily governs MSL complex assembly and proliferative signaling, whereas T418 phosphorylation may modulate catalytic efficiency or chromatin-associated activity in a more fine-tuning capacity. While these two sites may function cooperatively in regulating KAT8 activity, our current data also support the possibility that they exert partially independent regulatory effects. Importantly, global H4K16ac should be interpreted as a key downstream epigenetic readout of CDK1–KAT8 signaling, but not necessarily as the sole determinant of proliferation. Emerging evidence suggests that in mammalian somatic cells, H4K16ac influences higher-order chromatin organization and replication dynamics in addition to transcriptional activation [8,9,10,11]. Thus, additional CDK1-dependent pathways or locus-specific KAT8 functions may cooperate to generate the full proliferative phenotype.

CDK1 is traditionally recognized for driving the G2/M transition through Cyclin B binding, but it also regulates transcriptional and epigenetic programs through phosphorylation of chromatin-associated proteins such as P300, G9a, and Tip60 [27,28]. Here, we identify KAT8 as a previously unrecognized epigenetic substrate of CDK1. Although our biochemical assays primarily measure global H4K16ac, several observations argue against a purely non-specific global model. First, the T418A mutant reduces global H4K16ac but does not phenocopy the strong proliferative defect caused by S348A. Second, prior studies demonstrate that the KAT8-MSL complex is recruited to defined genomic loci to regulate specific transcriptional programs. Together, these findings raise the possibility that CDK-1-dependent stabilization of the MSL complex enhances H4K16ac at selected growth-promoting loci rather than uniformly across the genome. Nevertheless, definitive discrimination between locus-specific and global chromatin effects will require genome-wide approaches such as CUT&Tag or ChIP-seq analyses.

CDK1 is frequently overexpressed in multiple malignancies and correlates with poor clinical outcomes [29,30,31,32]. The selective CDK1 inhibitor RO-3306 [33] has shown antitumor efficacy in several preclinical models, including enhanced tumor suppression in combination with sorafenib in hepatocellular carcinoma [34], and synergistic impairment of homologous recombination repair in combination with PARP inhibitors in breast cancer [35]. In our study, RO-3306 suppresses lung cancer cell proliferation in association with reduced KAT8 phosphorylation at S348. Importantly, our conclusions are supported not only by pharmacological inhibition but also by complementary genetic approaches, including CDK1 knockdown and functional analysis of the KAT8-S348A mutant. These convergent strategies strengthen the specificity of the CDK1-KAT8 regulatory axis while acknowledging the inherent limitations of small-molecule inhibitors. Notably, wild-type KAT8 only partially rescues RO-3306-induced growth suppression. This partial rescue is informative, as it suggests that CDK1 promotes NSCLC proliferation through multiple downstream pathways. While the KAT8–H4K16ac axis represents a significant effector mechanism identified here, additional CDK1 substrates involved in cell cycle progression, transcription, or chromatin dynamics likely contributes in parallel.

Prior studies have shown that KAT8/MSL-mediated H4K16ac can directly activate transcription at specific loci [36], including growth-related genes such as *SKP2* in NSCLC [37]. In our xenograft model, RO-3306 reduces H4K16ac levels, and this effect is specifically rescued by re-expression of wild-type KAT8 but not by the S348A mutant. This allele-specific rescue supports a functional linkage between CDK1-mediated phosphorylation of KAT8 and downstream epigenetic output in vivo. However, we acknowledge that our data do not formally establish that global H4K16ac alone is sufficient to drive proliferation. Locus-specific chromatin events, or additional CDK1-dependent mechanisms may cooperate to produce the full tumor growth phenotype.

Finally, although our findings establish the CDK1–KAT8 axis as a driver mechanism in NSCLC, its relevance across solid tumors remains to be systematically investigated. Given the widespread dysregulation of CDK1 and KAT8 in diverse malignancies, this regulatory interaction may represent a broader epigenetic control node, though its impact is likely tumor-context dependent.

## 5. Conclusions

In summary, this study identifies CDK1 as a novel kinase that directly interacts with and phosphorylates KAT8 at residues S348 and T418. Mechanistically, CDK1-mediated phosphorylation, particularly at S348, promotes MSL complex assembly by enhancing the association between KAT8 and MSL1, thereby increasing KAT8 acetyltransferase activity toward H4K16. Functionally, the phosphorylation-deficient KAT8 S348A mutant fails to maintain MSL complex stability, leading to reduces H4K16 acetylation and suppressed NSCLC cell proliferation both in vitro and in vivo. Pharmacological inhibition of CDK1 with RO-3306 phenocopies these effects, and the anti-proliferative phenotype is partially rescued by reconstitution with wild-type KAT8 but not with the S348A mutant. Collectively, our findings uncover a previously unrecognized CDK1–KAT8 signaling axis that drives NSCLC progression through epigenetic regulation of H4K16ac. These results highlight CDK1-mediated phosphorylation of KAT8 as a potential therapeutic target for lung cancer intervention.

## Data Availability

The data that support the findings of this study are available from the corresponding author upon reasonable request.

## Abbreviations

The following abbreviations are used in this manuscript:

KAT8	Lysine acetyltransferase 8
CDK1	Cyclin-dependent kinase 1
H4K16ac	Histone H4 lysine 16 acetylation
CDKs	Cyclin-dependent kinases
NSCLC	Non-small cell lung cancer
PTMs	Post-translational modifications
HAT	Histone acetyltransferase
MSL	Male-specific lethal
